# Regulation of Seed Germination and Abiotic Stresses by Gibberellins and Abscisic Acid

**DOI:** 10.3389/fpls.2018.00838

**Published:** 2018-06-20

**Authors:** Bhushan Vishal, Prakash P. Kumar

**Affiliations:** Department of Biological Sciences, National University of Singapore, Singapore, Singapore

**Keywords:** gibberellins, abscisic acid, hormone signaling, seed germination, abiotic stresses, crosstalk of hormone signaling

## Abstract

Overall growth and development of a plant is regulated by complex interactions among various hormones, which is critical at different developmental stages. Some of the key aspects of plant growth include seed development, germination and plant survival under unfavorable conditions. Two of the key phytohormones regulating the associated physiological processes are gibberellins (GA) and abscisic acid (ABA). GAs participate in numerous developmental processes, including, seed development and seed germination, seedling growth, root proliferation, determination of leaf size and shape, flower induction and development, pollination and fruit expansion. Despite the association with abiotic stresses, ABA is essential for normal plant growth and development. It plays a critical role in different abiotic stresses by regulating various downstream ABA-dependent stress responses. Plants maintain a balance between GA and ABA levels constantly throughout the developmental processes at different tissues and organs, including under unfavorable environmental or physiological conditions. Here, we will review the literature on how GA and ABA control different stages of plant development, with focus on seed germination and selected abiotic stresses. The possible crosstalk of ABA and GA in specific events of the above processes will also be discussed, with emphasis on downstream stress signaling components, kinases and transcription factors (TFs). The importance of several key ABA and GA signaling intermediates will be illustrated. The knowledge gained from such studies will also help to establish a solid foundation to develop future crop improvement strategies.

## Introduction

Overall growth and different developmental stages of plants are under strict regulation by several classes of plant hormones. Hormone molecules are present at low concentrations in plants, and they function either at the sites of synthesis or after they are transported to different tissues (Santner et al., 2009; Li et al., 2016). In the last two decades, there has been rapid progress in the understanding of the biosynthetic pathways, transport, signaling and mode of action of various plant hormones. Studies related to hormone signaling have established the fact that besides acting on their own, various plant hormones interact in a highly intricate manner (Stamm et al., 2012; Kohli et al., 2013; Kumar, 2013a,b; Stamm and Kumar, 2013; Verma et al., 2015, 2016; Ravindran et al., 2017). These findings clearly indicate that plants maintain the availability and level of hormones in different parts of the plant body at different developmental stages in an intricate and balanced manner.

A convenient step for us to study plant growth may begin with seed germination. Successful germination depends on the ability of the plant embryo to gain its metabolic activity (Rajjou et al., 2012). Several molecular cues have been revealed by different genetic and proteomic investigations of various *Arabidopsis* mutants, showing distinct germination-related phenotypes (Achard et al., 2006, 2009; Magome et al., 2008). Germination has been found to be under strict regulation of plant hormones, including gibberellic acid (GA), abscisic acid (ABA), auxin and ethylene (Han and Yang, 2015). Germination is also significantly affected by several environmental factors, such as various abiotic stresses (Rajjou et al., 2012; Han and Yang, 2015). These factors mainly affect the metabolism and different signaling pathways of GA and ABA (Holdsworth et al., 2008).

The constantly changing external factors that most affect plant growth and development are abiotic stresses. Highly variable abiotic stresses affecting plant growth are salinity, drought, and cold. The above-mentioned stresses significantly affect yield (average yield reduction >50%) of crop plants (Mahajan and Tuteja, 2005). Plants exhibit a range of tolerance levels toward these stresses that are ultimately regulated by complex signaling pathways. Abiotic stresses trigger ABA biosynthesis, which mediates stress adaptive responses by activating several specific signaling cascades and regulating different physiological and growth-related processes.

In the past decade, several genetic, molecular and proteomic studies related to germination and abiotic stresses have been carried out. In this review, we discuss the roles of GA and ABA independently and with the possible crosstalk of these two phytohormones with respect to seed germination and abiotic stresses in various plant species, including crop plants.

## ABA and abiotic stress

Plants are capable of maintaining their internal environment fairly stable within the desired range. Two important factors that are crucial for the maintenance of such homeostasis are internal water level and osmotic state, which are mainly regulated by ABA (Zhang et al., 1987; Zhu, 2002). ABA acts as a molecular signal in response to various abiotic stresses, which alter the two important physiological functions mentioned above. These abiotic stress responsive signals are the basis of the various physiological as well as growth-related processes of plants, culminating in their unique ranges of tolerance toward these stresses (Finkelstein et al., 2002; Lee and Luan, 2012; Vu et al., 2015).

## ABA metabolism and abiotic stress

ABA which is reported in both primitive and higher organisms seems to have different biosynthesis pathways. In primitive organisms ABA biosynthesis is not well characterized, however, in plant-associated fungi, ABA is reported to be synthesized by the direct cytosolic pathway. In contrast, great progress has been made in identifying and characterizing the genes involved in ABA metabolism in land plants (Hauser et al., 2011). ABA biosynthesis in plants follows the organelle-specific indirect pathway. The pathway involves the key precursor compound zeaxanthin, which is synthesized by the β-carotene pathway involving pyruvate. Further, zeaxanthin is converted to xanthoxin by the enzymatic reaction catalyzed by ZEP enzyme (zeaxanthin epoxide) and 9-*cis*-epoxy carotenoid dioxygenase (NCED) enzyme (Hauser et al., 2011; Chan, 2012; Ruiz-Sola and Rodriguez-Concepcion, 2012). Subsequently, xanthoxin is transferred from the plastid to cytosol and converted to its aldehyde intermediate and then to ABA by short-chain-dehydrogenase reductase (SDR/ABA2 in *Arabidopsis*) and abscisic aldehyde oxidase (AAO), respectively (Cheng et al., 2002; González-Guzmán et al., 2014). Abiotic stresses and ABA treatment are reported to alter the transcript levels of key ABA biosynthesis genes, which in turn modulate the level of ABA in plants. Upon ABA treatment, expression levels of the genes encoding ZEP (*ZEP/ABA1/LOS6*) and AAO3 (*AAO3/ABA3/LOS5*) were upregulated in *Arabidopsis*. Furthermore, transcript levels of *NCED3, ABA3/LOS5*, and *AAO3* were induced by abiotic stresses (Xiong et al., 2002; Chan, 2012). Additionally, in crop plants improved tolerance toward various abiotic stresses has been reported by introducing or inducing expression of genes encoding key enzymes of ABA biosynthesis (Table 1). Among the NCED genes, *NCED3* expression level increased upon water stress, which is also reflected in the water-stress response of *nced3* mutants (Table 1).

**Table 1:** Summary of regulation of ABA metabolism and signaling genes with respect to abiotic stress and seed germination in different plant species.

	**Gene/protein encoded**	**Mutation/Overexpression**	**Plant studied**	**Effect on abiotic stress and/ or germination**	**Altered compounds/pathways/processes involved**	**References**
**I. ABA RELATED TRANSCRIPTION FACTORS**						
1.	*OsbZIP46CA1*	Overexpression	Rice	Drought tolerance	Positive regulator of ABA signaling	Tang et al., 2012
2.	*OsbZIP71*	Overexpression	Rice	Drought tolerance	Positive regulator of ABA signaling	Liu et al., 2014
3.	*OsbZIP52*	Overexpression	Rice	Cold and drought sensitivity	Negative regulator of ABA signaling	Liu et al., 2012
4.	*OsbZIP23*	Overexpression	Rice	Salinity and drought tolerance	Positive regulator of ABA signaling	Xiang et al., 2008
5.	*OsABF2*	Mutant	Rice	Sensitive to salinity, drought, and oxidative stress	Modulates transcript levels of abiotic stress-responsive genes	Hossain et al., 2010
6.	*DIG1* (Dynamic Influencer of Gene expression 1), *DIG2*	DEX Inducible expression	*Arabidopsis*	Salinity sensitivity	Differential ABA signaling	Song et al., 2016
7.	*OsAP2-39* (AP2-domain containing Transcription)	Overexpression	Rice	Low germination rate, ABA sensitivity, increased endogenous ABA level	Direct activation of ABA biosynthesis gene OsNCED1, whereas directly activating GA-inactivating gene OsEUI (Elongated Uppermost Internode)	Yaish et al., 2010; Shu et al., 2018
8.	*ORA47* (octadecanoid-responsive AP2/ERF-domain transcription factor 47)	Overexpression	*Arabidopsis*	Insensitive to wounding and water stress treatments, ABA insensitive	Direct regulation of ABA biosynthesis genes (NCED3 and NCED9) and the ABA-responsive gene RESPONSIVE TO DESICCATION 26 (RD26) under normal and wounding conditions but not under drought stress	Chen et al., 2016
9.	*ABA-INSENSITIVE 4 (ABI4)*	Mutant	*Arabidopsis*	Reduced primary seed dormancy, Resistant to paclobutrazol PAC (GA biosynthesis inhibitor)	ABI4 negatively regulates GA biosynthesis and by inhibits ABA catabolic genes expression (CYP707A1 and CYP707A2)	Shu et al., 2013, 2016
		Overexpression	*Arabidopsis*	Sensitive to PAC		
**II. ABA METABOLISM GENES**						
1.	*AtABA2* (Encodes ABA biosynthesis gene, short-chain-dehydrogenase reductase)	Overexpression	*Arabidopsis*	Delayed germination, Salinity tolerance	Increased ABA level, Altered primary metabolite level upon stress	Lin et al., 2007
2.	*SgNCED1* (from *Stylosanthes guianensis*)	Heterologous expression	Tobacco	Drought tolerance	Increase in the antioxidant enzyme activities	Bao et al., 2016
3.	*LeNCED1* (from tomato)	Heterologous expression	Petunia	Drought tolerance	Decreases in stomatal conductance, transpiration, and photosynthesis and increased concentrations of proline	Estrada-Melo et al., 2015
4.	Cytochrome P450 *CYP707A* encodes ABA 8′- hydroxylases	Mutant (*cyp707a2*)	*Arabidopsis*	Enhanced seed dormancy	Altered ABA levels in seeds	Asano et al., 2011
5.	AtBG1 (β-glucosidase)	Mutant (*atbg1*)	*Arabidopsis*	Dehydration sensitive and early germination	Defective stomatal movement	Lee et al., 2006
6.	AtBG2 (β-glucosidase)	Mutant (*atbg2*)	*Arabidopsis*	Dehydration and salinity sensitive	Altered ABA level	Xu et al., 2012
		Overexpression	*Arabidopsis*	Dehydration and salinity tolerance		
7.	BGLU10 (β-glucosidase)	Mutant (*bglu10*)	*Arabidopsis*	Drought sensitive	Increased rate of water loss, Reduced ABA content and expressions of ABA-and drought-responsive genes	Wang et al., 2011
		Overexpression	*Arabidopsis*	Drought tolerance	Reduced rate of water loss, Increased ABA content and expressions of ABA-and drought-responsive genes	
8.	AtDTX50 (DTX/Multidrug and Toxic Compound Extrusion (MATE) family member)	Mutant (*atdtx50*)	*Arabidopsis*	Drought tolerance	ABA efflux carrier in guard cells	Zhang et al., 2014
9.	AtPDR12/ABCG40 (a ATP-binding cassette (ABC) transporter)	Mutant (*atabcg40*)	*Arabidopsis*	Drought sensitive, Low rate of ABA-induced stomatal closer, impaired ABA regulation of seed germination and root development	Altered stomatal regulation, Altered cellular uptake of ABA	Kang et al., 2010
10.	AtABCG25 (a ATP-binding cassette (ABC) transporter)	Overexpression	*Arabidopsis*	less transpiration from the leaves (High leaf temperature)	AtABCG25 acts as an ABA exporter Which delivers ABA to guard cells.	Kuromori et al., 2010
11.	AtABCG22 (a ATP-binding cassette (ABC) transporter)	Mutant (*atabcg22*)	*Arabidopsis*	Drought sensitive, Increased transpiration through altered stomatal regulation	ABA signaling and ABA biosynthesis	Kuromori et al., 2011
12.	XERICO (encodes a RING-H2 zinc-finger protein)	Overexpression	*Arabidopsis*	Hypersensitivity to osmotic and salinity stress during germination and early seedling stage; and increased drought tolerance in the adult stage	Altered accumulation of ABA and differential expression GA, ethylene and ABA biosynthesis genes	Ko et al., 2006; Verma et al., 2016
			Rice	Salinity and drought tolerance	Increased ABA level and ABA-mediated stress response	Zeng et al., 2015
**III. ABA SIGNALING GENES**						
1.	*PYL8/RCAR3*	Overexpression	*Arabidopsis*	Hypersensitive to Salt and Osmotic Stresses	Positively regulates ABA signaling	Saavedra et al., 2010
2.	*PYL5* and *PYL7* (from tomato)	Heterologous expression	*Arabidopsis*	Drought tolerance	Not defined	González-Guzmán et al., 2014
3.	*SAPK9*	Overexpression	Rice	Drought tolerance	Modulating cellular osmotic potential, stomatal closure and stress-responsive gene expression	Dey et al., 2016
4.	*SnRKD,E,I*	*srk2d/e/i* triple mutant	*Arabidopsis*	Drought sensitivity	Decreased expression of ABA- and stress-inducible genes	Fujita et al., 2009
5.	*ZmSAPK8* (from corn)	Heterologous expression	Arabidopsis	Salinity tolerance	Proline accumulation and low relative electrolyte leakage	Ying et al., 2011
6.	*Abscisic acid-insensitive(abi)*	Mutant (*abi3, abi4, and abi5*)	*Arabidopsis*	ABA-mediated inhibition of seed germination and low water potential-induced ABA and proline accumulation	Altered ABA or Proline accumulation	Finkelstein, 1994; Verslues and Bray, 2006
7.	*SIZ1* (SUMO E3 ligase)	Mutant (*siz1–2* and *siz1–3*)	*Arabidopsis*	Increased ABA inhibition of seed germination and seedling primary root growth.	Induced expression of genes that are ABA-responsive (ABI5-dependent signaling) (e.g., RD29A, Rd29B, AtEm6, RAB18, ADH1)	Miura et al., 2009

The expression of ABA biosynthesis genes is reported to show a direct impact on seed germination along with abiotic stresses. The identification and characterization of NCED genes revealed that the tissue-specific expression of these genes and the resultant modulation of endogenous ABA level at different developmental stages are responsible for the regulation of specific processes, such as seed maturation and seed germination, besides response to abiotic stresses (Lefebvre et al., 2006; Martínez-Andújar et al., 2011). Within the seeds, *NCED6* was shown to express in the endosperm whereas *NCED9* is expressed in both embryo and endosperm during *Arabidopsis* seed development. The induction of *NCED6* inhibits seed germination by increasing the endogenous level of ABA. These and similar findings have clearly established a causal role for ABA in regulating the physiological and developmental processes studied.

It is known that ABA accumulates under specific conditions, such as abiotic stresses. Therefore, the endogenous concentration of biologically active ABA at the site of perception has to be regulated. Apart from biosynthesis, ABA catabolism and transport are the two key essential processes that control ABA-mediated stress regulation. Cytochrome P450 type enzymes (CYP707As) catalyze the deactivation reaction resulting in phaseic acid (PA) and dihydro phaseic acid (DPA) as the main ABA catabolites (Ng et al., 2014; Sah et al., 2016), which do not appear to have any significant biological activity (Sharkey and Raschke, 1980; Kepka et al., 2011). ABA and its catabolites (hydroxylated) can be conjugated to glucose, catalyzed by ABA glucosyl ester (ABA-GE) and become inactivated (Zeevaart and Creelman, 1988; Lim et al., 2005). However, ABA-GE could be converted to ABA upon induction of different abiotic stresses (Ye et al., 2012; Sah et al., 2016). Two β-glucosidases, AtBG1 and AtBG2 localized in the vacuole and endoplasmic reticulum, respectively, hydrolyze ABA-GE (Burla et al., 2013). ABA is a weak acid (pKa ~ 4.7), which can be protonated to become membrane permeable so that it can diffuse passively across the cell membrane (Wilkinson and Davies, 2010; Ng et al., 2014; Sah et al., 2016). Several transporters have been identified in different species of plants, which regulate the accumulation and translocation of active ABA along the plant body involving different organelles (Kang et al., 2010; Kanno et al., 2012; Ye et al., 2012). Also, several genes related to ABA metabolism and transport in different plant species are reported to alter abiotic stress tolerance summarized in Table 1.

## ABA signaling genes, abiotic stress, and germination

The identification of ABA receptors in Arabidopsis and other plant species is one of the key findings in ABA signaling. The PYR/PYL/RCAR family of proteins are established as the most plausible ABA receptors. Expression profile study of these receptors revealed their role in ABA signaling as well as in the regulation of abiotic stresses (Park et al., 2009). Triple and quadruple mutants of *pyl* showed altered ABA sensitivity with respect to seed germination and growth, while overexpression lines conferred tolerance toward abiotic stress (Santiago et al., 2009; Saavedra et al., 2010). Overexpression of *RCAR* gene resulted in altered ABA-dependent germination and seedling growth (Ma et al., 2009). PYR/PYL/RCAR receptors in the presence of ABA form a complex and deactivate PP2C, which otherwise inactivates the SnRK2s, a central regulator of ABA signaling. Subclass III of SnRK2 in *Arabidopsis* and rice are shown to be involved in ABA signaling (Kobayashi et al., 2005). Their expressions were induced in the presence of ABA. Furthermore, they are responsible for the activation several ABRE binding factors (ABFs). ABFs belong to basic leucine zipper (bZIP) transcription factor family, which is one of the key regulators of ABA responses in plants. In general, they interact with the *cis*-acting conserved regulatory element, ABREs (ABA-responsive elements) and in turn regulate transcription of several downstream ABA-responsive genes (Choi et al., 2000; Kim et al., 2002; Lopez-Molina et al., 2002). Table 1 summarizes the effect of the genes related to ABA signaling with respect to various abiotic stresses in different plant species.

## GA biosynthesis genes, abiotic stress, and seed germination

The discovery of bioactive gibberellic acid (GA) was the result of an investigation of fungal (*Gibberella fujikuroi*) infection in rice by Teijiro Yabuta and co-workers (Yabuta and Sumiki, 1938). Since then, more than a hundred GAs have been identified from different sources, (from bacteria to plants). However, only a few of them have been shown to have biological activity (Yamaguchi, 2008; Hedden and Thomas, 2012). Gibberellins control different stages of plant development, including seed germination, seedling growth, stem elongation, root extension, leaf size and shape, flower and fruit development, pollination (García-Martínez et al., 1997; Yamaguchi, 2008; Hedden and Thomas, 2012).

In plants three classes of enzymes are required for the biosynthesis of bioactive GAs (GA_1_, GA_3_, and GA_4_) from the precursor compound geranylgeranyl diphosphate (GGDP), which is aided by terpene synthases (TPSs), cytochrome P450 monooxygenases (P450s), and 2-oxoglutarate–dependent dioxygenases (2ODDs) (Yamaguchi, 2008; Hedden and Thomas, 2012). Two TPSs, *ent*-copalyl diphosphate synthase (CPS) and *ent*-kaurene synthase (KS), which are located in the plastids are responsible for the first few steps of GA biosynthesis (conversion of GGDP to *ent*-kaurene). Then two P450 enzymes, namely, *ent*-kaurene oxidase (KO) and *ent*-kaurenoic acid oxidase (KAO) convert *ent*-kaurene to GA_12_. Finally, three active GAs are formed by reactions catalyzed by GA 20-oxidase (GA20ox) and GA 3-oxidase (GA3ox), that belong to 2ODDs (Yamaguchi and Kamiya, 2000; Hedden, 2001; Yamaguchi, 2008; Hedden and Thomas, 2012). In plants, deactivation of the GAs is critical for maintaining the levels of bioactive GAs, which is regulated by GA 2-oxidases (GA2oxs), belonging to 2ODDs (Yamaguchi and Kamiya, 2000; Yamaguchi, 2008). Additionally, 16α,17-epoxidation (Luo et al., 2006; Zhu et al., 2006) and methylation of the C-6 carboxyl group of GAs (Varbanova et al., 2007) are involved in the deactivation of GAs in different plant species.

Several GA biosynthesis genes are expressed in growing tissues during *Arabidopsis* development (Silverstone et al., 1997) and also in crop plants such as wheat (Aach et al., 1997), rice (Kaneko et al., 2003), and tobacco (Itoh et al., 1999). This suggests that biologically active GAs are synthesized at the site of their action in several cases. However, in rice, it has been shown that GA biosynthesis genes are not expressed in the aleurone layer, but GA signaling event occurs there, which suggests paracrine signaling by GAs (Kaneko et al., 2002, 2003). In addition, in *Arabidopsis*, GA-dependent gene expressions have been shown in the sites where bioactive GAs are not produced (Yamaguchi et al., 2001). It has also been shown that early and late steps of GA biosynthesis take place in provascular tissue and, cortex and endodermis, respectively (Yamaguchi and Kamiya, 2000; Yamaguchi et al., 2001). This suggests the existence of intercellular movement/transport of GA biosynthesis intermediates. Lack or absence of GA leads to altered GA signaling and germination related phenotype, which has been revealed by different studies done in mutants of GA metabolism (Table 2). The relationship between expressions of GA metabolism-related genes and tolerance toward abiotic stresses have been shown. Mutants in GA biosynthesis genes (*GA20ox* and *GA3ox*) showed drought tolerance phenotype and overexpression of *GA20ox* confers drought sensitivity in *Arabidopsis* (Colebrook et al., 2014).

**Table 2:** Summary of regulation of GA metabolism and signaling genes with respect to abiotic stress and seed germination in different plant species.

	**Gene**	**Protein encoded**	**Genotype**	**Plant studied**	**Effect on abiotic stress and/ or germination**	**Altered compound/pathways/processes**	**References**
**I. GA METABOLISM AND REGULATORS OF GA SIGNALING**							
1.	*GAI, RGA, RGL1*, and *RGL2*	DELLA Protein	DELLA quadruple-mutant *gai-t6 rga-t2 rgl1-1 rgl2-1*	*Arabidopsis*	Salt sensitivity, Early flowering	DELLA-dependent reduced accumulation of bioactive GAs	Achard et al., 2006
2.	GA-INSENSITIVE (*GAI*)	DELLA Protein	Mutant (*gai*)	*Arabidopsis*	Salinity tolerance	DELLA-dependent growth restraint	Achard et al., 2006
3.	GA-deficient *GA1-3*	DELLA Protein	Mutant (*ga1-3*)	*Arabidopsis*	Salinity tolerance	DELLA-dependent growth restraint	Achard et al., 2006
4.	*PROCERA*	DELLA Protein	Mutant (*pro*)	Tomato	Enhanced stomatal conductance and rapid wilting under drought stress	Negative regulator of GA signaling	Nir et al., 2017
5.	NUCLEAR FACTOR Y C proteins (NF-YC) homologs *NF-YC3, NF-YC4* and *NF-YC9* Nuclear factor Y (NF-Y) family proteins		Mutant *nf-yc3 nf-yc4 nf-yc9* (*nf-ycT*)	*Arabidopsis*	Higher germination rates than the wild type in the presence of a of paclobutrazol PAC (GA biosynthesis inhibitor)	Altered GA and ABA signaling	Liu et al., 2016
			Overexpression (*35S:NF-YC3* and *35S:NF-YC9*)	*Arabidopsis*	Lower germination rates than the wild type in the presence of a of PAC		
6.	*GA1-3* and *GA2*	GA biosynthesis genes	Mutant (*ga1-3 and ga2*)	*Arabidopsis*	Unable to germinate in the absence of exogenous GA	Absence or the changed composition of endogenous GA's	Koornneef and Van Der Veen, 1980
7.	*GIB-1*	Gibberellin biosynthesis gene	Mutant (*gib-1*)	Tomato	Unable to germinate without exogenous GA	Lack of GA	Karssen et al., 1989; Benson and Zeevaart, 1990
8.	*GID1, GID2*, and *GID3*	GA INSENSITIVE DWARF (GID) GA receptor	Mutant (*gid1a gid1b gid1c*)	*Arabidopsis*	Unable to germinate	The absence of DELLAs destruction by gibberellins	Willige et al., 2007
9.	*DAG1*	Transcription factor DOF AFFECTING GERMINATION 1 (DAG1)	*dag1*	*Arabidopsis*	Reduced GA requirement for the seeds to germinate	Negative regulation GA biosynthesis by acting downstream of PIL5 (PHYTOCHROME INTERACTING FACTOR 3-LIKE 5)	Gabriele et al., 2010
10.	*SPY-1* and *SPY-3*	O-linked N-acetylglucosamine transf Erase	Mutants (*spy-1 and spy-3*)	*Arabidopsis*	Drought and salinity tolerance	Altered environmental stress signals via Gibberellin and Cytokinin cross talk	Qin et al., 2011
			Overexpression	*Arabidopsis*	Drought sensitive		
11.	*KYP/SUVH4*	Histone methyltransferase	Mutant	*Arabidopsis*	Increased seed dormancy and sensitivity to ABA	Altered ABA and GA regulation, and altered expression of dormancy-related genes	Zheng et al., 2012
			Overexpression	*Arabidopsis*	Reduced seed dormancy and sensitivity to ABA		
**II. GA-RELATED TFS**							
1.	*DDF1*	AP2 transcription factor of the DREB1/CBF subfamily	Overexpression	*Arabidopsis*	Salinity tolerance	Upregulates expression of a gibberellin-deactivating gene, GA2ox7	Magome et al., 2008
2.	*ERF6*	Ethylene response factor (ERF)	Gain-of-function	*Arabidopsis*	Hypersensitive to osmotic stress	Inhibits growth through a GA/DELLA dependent mechanism	Dubois et al., 2013
3.	*CBF1*	C-repeat/drought-responsive element binding factor	Overexpression	*Arabidopsis*	Cold tolerance, Inhibit seed germination	Accumulation of DELLAs	Achard et al., 2008
4.	*PIL5*	PHYTOCHROME INTERACTING FACTOR 3-LIKE 5	Overexpression	*Arabidopsis*	Inhibits seed germination	Activation of a GA catabolic gene (*GA2ox2*) and repression of GA biosynthesis genes *GA3ox1* and *GA3ox2*	Oh et al., 2007
5.	*CHOTTO1*	Double APETALA2 Repeat Transcription Factor	Overexpression	*Arabidopsis*	Sensitive to ABA during seed germination processes	Inactivates GA biosynthesis	Yano et al., 2009; Shu et al., 2018
6.	SNORKEL1 and SNORKEL2	Ethylene response factor (ERF) domain proteins	Overexpression	Rice	Submergence-tolerance	Increases in bioactive GA levels	Hattori et al., 2009
7.	SUB1A	Ethylene response factor (ERF) domain proteins	Overexpression	Rice	Submergence-tolerance	Negatively regulates GA responses by accumulation of the GA signaling repressors Slender Rice-1 (SLR1) and SLR1 Like-1 (SLRL1)	Fukao and Bailey-Serres, 2008

**Figure 1:**
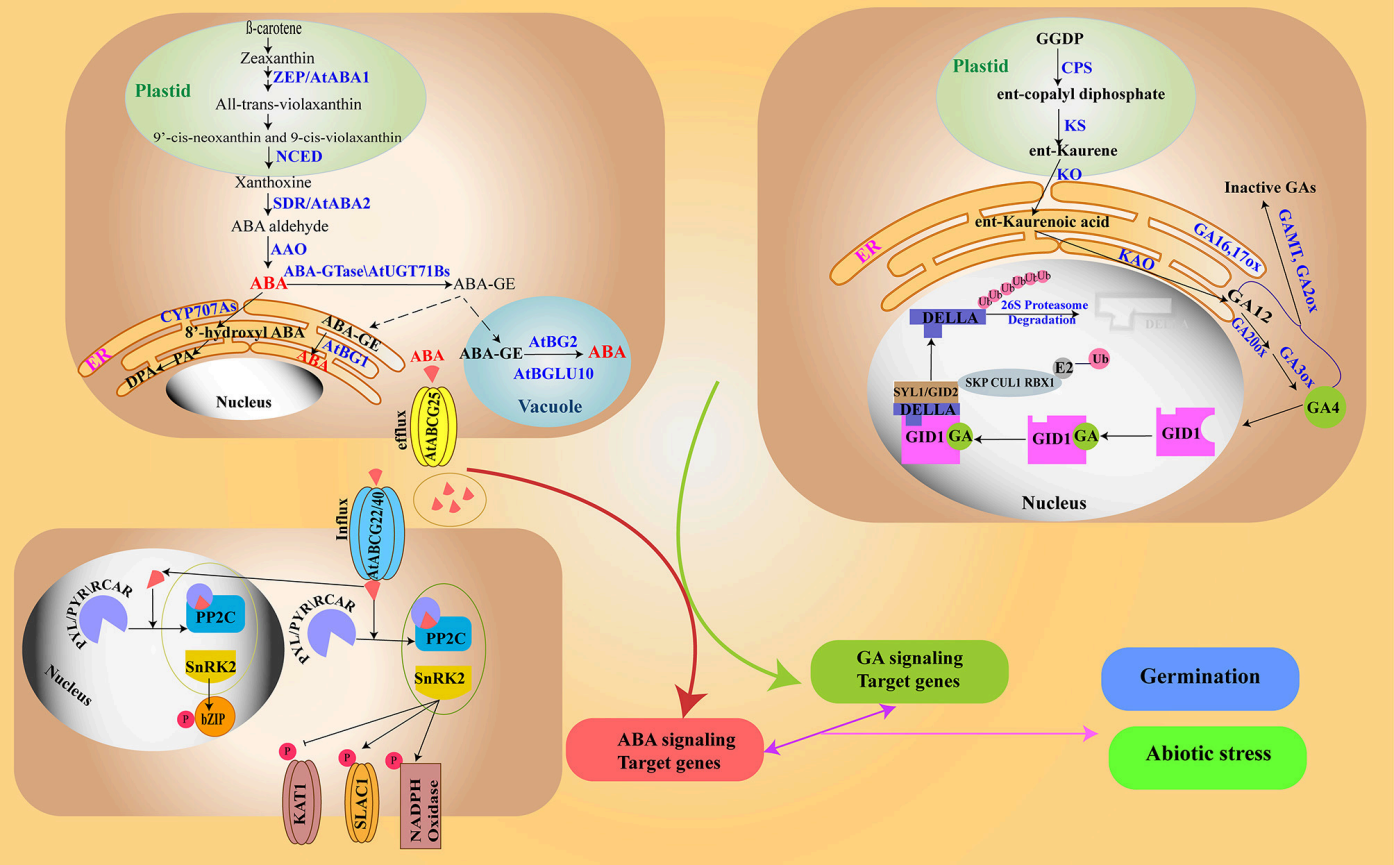
ABA and GA metabolism and signaling/ABA is synthesized from carotenoids in a series of reactions in the plastids and cytoplasm (top left). ABA is catabolized to form phaseic acid. ABA transport occurs through different transporters, and ABA elicits distinct signaling cascades (in the nucleus and cytoplasm) (bottom left). GA biosynthesis starts from GGDP in the plastid and a portion of it is catabolized to inactive forms (top right). In the GA signaling pathway, GA causes destruction of DELLAs (negative regulator of GA) via the 26S proteasome machinery. The two signaling pathways crosstalk to regulate seed germination and abiotic stresses (bottom right).

Characterization of mutants and genetic studies revealed several GA signaling components (Hedden and Phillips, 2000; Stamm et al., 2012; Davière and Achard, 2013). DELLA proteins, belonging to the GRAS family of transcription factors, are identified as a major repressor of GA signaling. DELLA proteins restrict cell proliferation and expansion by negatively regulating gibberellin signaling and hence inhibit the plant growth (Peng et al., 1997, 1999; Fleet and Sun, 2005). DELLA proteins such as RGL2 complexed with DOF6 transcription factor has also been shown to have positive effect on target genes such as *GATA12* in regulating seed germination (Ravindran et al., 2017). DELLAs are degraded by a signal cascade involving GA and its positive regulators (Hedden and Phillips, 2000; Achard and Genschik, 2009; Wang et al., 2009; Figure 1). The GA signaling components are reported to affect various aspects of germination and abiotic stresses as well (Table 2). DELLAs are also reported to confer salt tolerance in *Arabidopsis* by altering the duration of vegetative growth. Also, two of the DELLA proteins, RGA and GAI have a major role in salt-induced plant growth regulation (Achard et al., 2006).

## GA and ABA crosstalk

Hormones regulate plant growth and development either synergistically or antagonistically, involving a series of complex pathways and networks (Liu et al., 2010; Dong et al., 2016; Rowe et al., 2016). In the preceding sections, we described the individual roles of GA and ABA in two important aspects affecting plant development; germination and abiotic stresses. The information summarized in Tables 1, 2 along with the preceding description show that ABA and GA antagonistically mediate plant developmental processes including seed dormancy and germination. Hence, it is essential to maintain an optimal balance between the endogenous levels of ABA and GA for plant development.

In response to different developmental stages and environmental conditions, various changes occur in the metabolism and signal transductions of these two plant hormones which keep a correct balance between GA and ABA and hence plant homeostasis. In the following sections we will summarize how genes, components and network involving crosstalk of GA and ABA participate in the regulatory processes.

In many instances, possible crosstalk events have been shown between ABA and GA with respect to various abiotic stresses and plant growth. Unfavorable conditions lead to high ABA and low GA levels in seeds whereas favorable conditions cause the reverse situation. Seed dormancy is maintained by ABA whose level is found to progressively increase from embryogenesis to embryo maturation (Karssen et al., 1983). ABA restricts embryo growth potential by inhibiting water uptake (imbibition) and hence cell-wall loosening, which is a key step to start germination (Schopfer and Plachy, 1984; Gimeno-Gilles et al., 2009). ABA also leads to induction of Late Embryogenesis Abundant (*LEA*) genes and growth arrest by activating a basic leucine zipper transcription factor, ABSCISIC ACID INSENSITIVE 5 (ABI5) (Finkelstein and Lynch, 2000). Many *LEA* genes are reported to confer abiotic stress tolerance in plants (Lopez-Molina and Chua, 2000; Lee et al., 2005). Synergistic repression of germination has been reported through ABRE and RY elements by ABI5 and ABI3 (activated by ABA) (Lopez-Molina et al., 2002; Park et al., 2011). Under favorable conditions (light, temperature and moisture) GA biosynthesis and associated pathways are activated, which results in the release from the inhibitory effect of ABA. Cold stratification and light lead to an increase in bioactive GAs via transcription factors PIF3-like 5 (PIL5), Blue Micropylar End3 (BME3) and SPATULA (SPT) (Liu et al., 2005; Penfield et al., 2005; Oh et al., 2006; Figure 1). Thus, it is clear that various interactions between ABA and GA in seeds help to regulate dormancy and germination.

Several recent studies showed the regulation of GA and ABA in light- and temperature-mediated seed germination and dormancy. PIL5, a light-labile transcription factor, regulates both GA and ABA signaling and thereby inhibits seed germination. It indirectly regulates GA biosynthesis genes and directly regulates GA signaling genes. Thus, PIL5 represses GA biosynthesis genes (*GA3ox1* and *GA3ox2*) and activates a GA catabolic gene (*GA2ox2*) indirectly (Gabriele et al., 2010). However, it binds to the promoter region of the GA signaling repressor genes, *GAI* and *RGA* and regulates their transcription (Oh et al., 2007). On the other hand, PIL5 has the opposite effect on the ABA biosynthesis genes. It activates ABA biosynthesis genes (*ABA1, NCED6*, and *NCED9*) and represses an ABA catabolic gene (*CYP707A2*) (Finkelstein et al., 2008). Furthermore, increased expression of *DELAY OF GERMINATION 1* (*DOG1*) which acts downstream to PIL5, leads to repression of GA biosynthesis and activation of *ABI3* and *ABI5* (Bentsink et al., 2006; Skubacz and Daszkowska-Golec, 2017). Similarly, a CCCH-Type zinc finger protein, SOMNUS (SOM) is reported to act downstream of PIL5 in order to negatively regulate light-dependent seed germination in *Arabidopsis* (Kim et al., 2008). Several other CCCH zinc finger proteins (AtTZF4, 5, and 6) negatively regulate GA- and light-mediated seed germination and positively regulate ABA-mediated seed germination. Expression patterns of genes regulating GA and ABA metabolism have been reported to be well coordinated with seasonal seed dormancy in *Arabidopsis*. Thus, upregulation of GA catabolism and ABA biosynthesis genes was observed during low temperature (winter) which leads to increased dormancy (Footitt et al., 2011). Consistent with that, upregulation of GA biosynthesis ABA catabolism genes have been reported during high temperature (spring and summer) and decreased dormancy (Footitt et al., 2011). The transcription factor SPT controls the germination response to cold and light. It can repress the GA biosynthesis genes (*GA3ox1* and *GA3ox2*) (Penfield et al., 2005) as well as the expression of *ABI4* and a DELLA gene *RGA*, but it promotes expression of *ABI5* and *RGL3*, another DELLA gene (Vaistij et al., 2013).

Various abiotic stresses (external environment) lead to changes in the plant response and therefore alter the balance of endogenous levels of GA and ABA. High temperature induces ABA biosynthesis genes (*ZEP, NCED2, NCED5*, and *NCED9*) and hence increases the ABA level whereas it decreases the GA level by repressing GA biosynthesis genes in *Arabidopsis* seeds (Toh et al., 2008). The transcription factor FUS3 leads to delayed germination at high temperature by activating seed-specific, ABA biosynthetic and ABA signaling genes (Chiu et al., 2012). ABI3, ABI5, and DELLAs form a complex to directly activate *SOM* expression at high temperature, which results in altered expression of ABA and GA metabolism genes (Lim et al., 2013).

**Figure 2:**
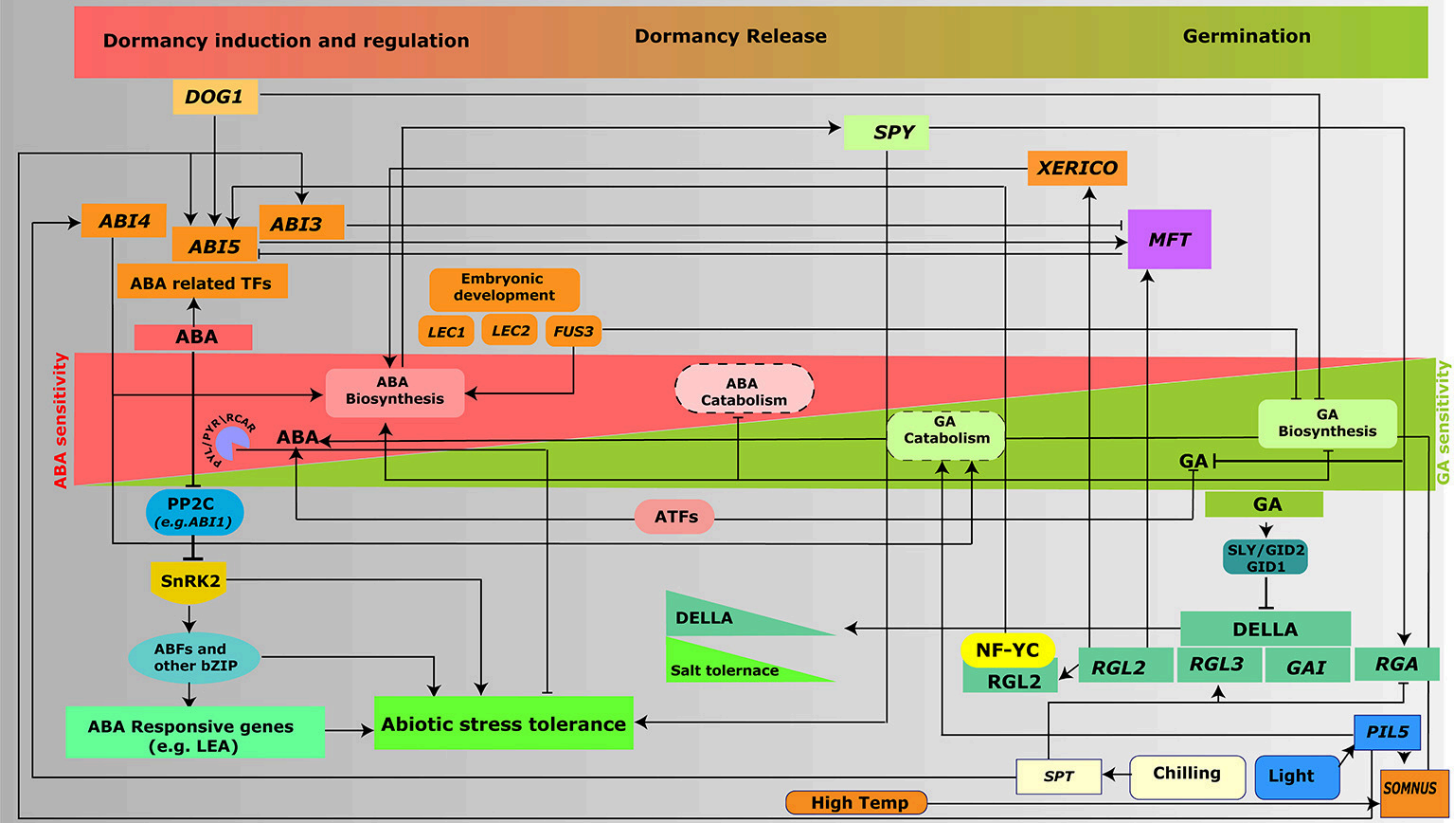
Interplay of ABA and GA signaling in the regulation of seed germination and abiotic stresses. Switch from seed dormancy to germination is controlled by the intricate balance between ABA and GA levels. ABA- and GA-signaling and metabolism genes regulate the expression of various genes (as mentioned in the text) and hence control two of the major aspects of plant development, germination and response to abiotic stresses.

DELLA-dependent salt-induced growth inhibition in the DELLA quadruple-mutant was also shown to be associated with DELLA accumulation and ABA signaling. Also, upon ABA treatment accumulation of GFP-RGA was not observed in the *abi1-1* roots, but only seen in the untreated WT control (Peng et al., 1997; Fleet and Sun, 2005; Achard et al., 2006), showing the crosstalk between ABA and GA signaling. Furthermore, quadruple-DELLA mutant was also shown to have ABA insensitive phenotype. In addition, ABA-induced delay in flowering was shown to be DELLA dependent (Achard et al., 2006). PROCERA (a DELLA protein in tomato) promotes stomatal closure in an ABA-dependent manner by increasing ABA sensitivity (Nir et al., 2017). Another study showed that NUCLEAR FACTOR-Y C (NF-YC) homologs (NF-YC3, NF-YC4, and NF-YC9) interact with the DELLA protein RGL2 and target ABI5 (Liu et al., 2016), thus regulating germination by modulating GA- and ABA-responsive genes in *Arabidopsis*. In addition, NF-YC9 was also reported to regulate ABA signaling via direct interaction with ABI5 (Bi et al., 2017). Therefore, NF-YC family members could integrate GA and ABA antagonistic crosstalk involving DELLA protein and ABA signaling TFs. Global analysis of DELLA targets revealed several downstream targets and responsive genes (Zentella et al., 2007). *XERICO* which has a key role in mediating various abiotic stresses by modulation of ABA level and expression of ABA-responsive genes is a target of DELLA (Ko et al., 2006; Zentella et al., 2007; Zeng et al., 2015). Other reports have also shown that DELLA contributes toward upregulation of ABA level by increasing the *XERICO* transcript levels (Zentella et al., 2007; Figure 2). This represents another example of how DELLA proteins can control plant growth and abiotic stress tolerance through specific crosstalk with ABA signaling pathway.

DELLA repressors are mainly degraded through the ubiquitin-proteasome system involving recruitment of Skip, Cullin, and F-box E3 ubiquitin ligase to the GA-GID1-DELLA complex by SLEEPY1 (SLY1) (Steber et al., 1998; Hedden, 2001; Murase et al., 2008; Achard and Genschik, 2009; Wang et al., 2009; Figure 1). In addition to ubiquitination, the DELLA signaling components are regulated by SUMOylation (small ubiquitin-related modifier). E3 SUMO ligase AtSIZ1 negatively regulates ABA signaling by SUMOylation of ABI5 in *Arabidopsis* during germination (Miura et al., 2009; Liu and Hou, 2018). In addition, AtSIZ1 was reported to positively regulate GA signaling by SUMOylating SLY1 (Kim et al., 2015; Liu and Hou, 2018). Therefore, SIZ1 could be another direct link between GA and ABA signaling by regulating ABI5 and SLY1.

Another E3 ligase, ANAPHASE-PROMOTING COMPLEX/CYCLOSOME (APC/C) has a link between GA and ABA signaling in rice via SnRK2-APC/C^TE(Tiller Enhancer/activator)^ module (Lin et al., 2015; Liu and Hou, 2018). Loss-of-function of TE, leads to hyposensitivity to GA and hypersensitivity to ABA. Furthermore, ABA inhibits APC/C^TE^ activity by phosphorylation of TE through the activation of rice SnRK2s. This event interrupts the association between TE and OsPYL/RCARs (ABA receptor), which results in stabilization of the receptor. Conversely, opposite effect has been shown by GA by inhibiting rice SnRK2 (Lin et al., 2015).

Several TFs other than DELLAs have been reported to act as potential mediators between ABA and GA metabolism and signaling. Two APETALA 2 (AP2)-domain containing transcription factors (ATFs), Arabidopsis ABA-INSENSITIVE 4 (ABI4) and rice OsAP2-39, have key roles in the antagonistic crosstalk between ABA and GA. ABI4 positively regulates primary seed dormancy by downregulating GA biosynthesis and by inhibiting ABA catabolic genes (*CYP707A1* and *CYP707A2*) (Shu et al., 2013). Further, GA represses the expression level of ABA biosynthesis gene, *NCED6* and increases expression of the GA-deactivating gene *GA2ox7* in an ABI4-dependent manner (Shu et al., 2016). In rice, OsAP2-39 induces ABA level by directly activating ABA biosynthesis gene *OsNCED1*, whereas it reduces GA level by directly activating GA-inactivating gene *OsEUI* (Elongated Uppermost Internode) (Shu et al., 2018). Further, enhanced ABA level due to activation of *OsNCED1* induces the *OsEU1* expression, which ultimately decreases GA accumulation (Yaish et al., 2010; Shu et al., 2018). Another study showed that CHOTTO1, a double-AP2 domain-containing TF regulates seed germination in *Arabidopsis* through ABA-mediated repression of GA biosynthesis (Yano et al., 2009).

MYB96 TF controls primary seed dormancy by directly activating ABA biosynthesis genes (*NCED2, NCED5, NCED6*, and *NCED9*) and indirectly repressing GA biosynthesis genes (*GA3ox1* and *GA20ox1*) (Lee et al., 2015). Another key regulator of seed dormancy Mother of FT and TFL 1 (MFT), controls ABA and GA signaling pathways (Xi et al., 2010). MFT promotes germination by downregulating ABA signal via repression of *ABI5* expression. MFT expression is induced by RGL2 and ABI5, but downregulated by ABI3 and MFT (Xi et al., 2010; Skubacz and Daszkowska-Golec, 2017; Figure 2). Another three transcriptional regulators involved in regulating embryonic development are the *LEC* genes; *LEAFY COTYLEDON1* (*LEC1*), B3 domain factors *LEC2*, and *FUSCA3* (*FUS3*) (Keith et al., 1994; Gazzarrini et al., 2004). Loss-of-function of these genes leads to the alteration of embryonic leaves (cotyledons) to take on the appearance of vegetative leaves (Gazzarrini et al., 2004). One of them, *FUS3* is known to positively regulate ABA biosynthesis, and negatively regulate GA biosynthesis (Gazzarrini et al., 2004). Another B3 TF GERMINATION DEFECTIVE 1 (GD1) regulates seed germination by suppressing a *LEC2/FUS3*-like gene of rice (*OsLFL1*) and modulating expression of GA metabolic genes (*OsGA3ox, OsGA20ox*, and *OsGA2ox*) (Guo et al., 2013).

These examples clearly show the crosstalk between ABA and GA in controlling seed development as well as germination. Such crosstalk has been predicted based on earlier studies. With the limited number of definitive studies on such signal crosstalk, we are just beginning to gain valuable insights regarding the regulation of specific growth and developmental processes.

## Conclusions and future perspectives

It is evident from the foregoing review that the signaling interactions among several phytohormones are common in regulating various stages and processes of plant development. Such regulatory crosstalk can occur at multiple stages of biosynthesis or signaling for different hormones. Biosynthesis of bioactive hormones and their transport (passive and/or active) as well as signaling cascades that regulate downstream target genes (of different classes) further add to the complexity of the already elaborate cellular communication network. This has been highlighted here with the examples of ABA and GA metabolism and their regulation. Selected genes that play significant roles in the regulation of seed dormancy and germination and various abiotic stresses were also discussed. It is evident that several positive and negative regulators of ABA and GA have direct or indirect impacts on germination and abiotic stresses. Many transcription factors and signaling components of these two phytohormones help to maintain an intricate balance between endogenous levels of bioactive ABA and GA. Furthermore, studies have identified several ABA and GA crosstalk points showing positive and negative regulation of different molecular modules associated with their metabolism and signaling. There are a few open questions that can help in formulating the future research directions. Despite the fact that there are some studies on ABA transport in different cell types and tissues, there might be many unknown pathways/transporters that are yet to be explored. Moreover, very few reports on the transport mechanism of GA are available. The antagonistic roles of GA and ABA in controlling developmental processes have been established by several pieces of evidence; however, there could be synergistic crosstalk between GA and ABA in some instances whose underlying molecular mechanisms remain undiscovered. Although several target genes of a few TFs have been established (eg. MYB96, ABI4, OsAP2-39) (Yaish et al., 2010; Shu et al., 2013; Lee et al., 2015) identification of direct targets/genes of several TFs and components of GA and ABA signaling modules are worth investigating. The detailed analyses of direct targets involved in GA and ABA metabolism and signaling at different developmental stages will provide us with more insights into GA and ABA crosstalk. Recent studies revealed several new cues associated with GA and ABA signaling. A few epigenetic modifiers have been documented to be involved in GA and ABA signaling cascade (Ryu et al., 2014; Liu et al., 2016; Peirats-Llobet et al., 2016). However, the mechanisms by which these epigenetic regulators mediate crosstalk between GA and ABA need to be investigated. It is known that complexes of TFs regulate downstream target genes (Kepka et al., 2011; Lim et al., 2013; Heyman et al., 2016; Iwata et al., 2017), and therefore, future investigations into new protein complexes associated with GA and ABA signaling will reveal interesting molecular mechanisms of developmental regulation. Although several signaling components controlling various aspects of germination and abiotic stresses have been identified, the nature of the underlying mechanisms of many of the events remain to be clarified. Nevertheless, such specific interaction points that have been identified for these two phytohormones will offer potential genetic intervention strategies to control growth and abiotic stress remediation in future crop breeding programs.

## Author contributions

BV and PK conceived the idea and wrote the manuscript.

### Conflict of interest statement

The authors declare that the research was conducted in the absence of any commercial or financial relationships that could be construed as a potential conflict of interest.

## References

[B1] Aach H. Bode H. Robinson D. G. Graebe J. E. (1997). ent-Kaurene synthase is located in proplastids of meristematic shoot tissues. Planta 202, 211–219. 10.1007/s004250050121

[B2] Achard P. Cheng H. De Grauwe L. Decat J. Schoutteten H. Moritz T.. (2006). Integration of plant responses to environmentally activated phytohormonal signals. Science 311, 91–94. 10.1126/science.1118642 16400150

[B3] Achard P. Genschik P. (2009). Releasing the brakes of plant growth: how GAs shutdown DELLA proteins. J. Exp. Bot. 60, 1085–1092. 10.1093/jxb/ern301 19043067

[B4] Achard P. Gong F. Cheminant S. Alioua M. Hedden P. Genschik P. (2008). The cold-inducible CBF1 factor–dependent signaling pathway modulates the accumulation of the growth-repressing DELLA proteins via its effect on gibberellin metabolism. Plant Cell 20, 2117–2129. 10.1105/tpc.108.058941 18757556 PMC2553604

[B5] Achard P. Gusti A. Cheminant S. Alioua M. Dhondt S. Coppens F.. (2009). Gibberellin signaling controls cell proliferation rate in *Arabidopsis*. Curr. Biol. 19, 1188–1193. 10.1016/j.cub.2009.05.059 19576768

[B6] Asano T. Hakata M. Nakamura H. Aoki N. Komatsu S. Ichikawa H.. (2011). Functional characterisation of OsCPK21, a calcium-dependent protein kinase that confers salt tolerance in rice. Plant Mol. Biol. 75, 179–191. 10.1007/s11103-010-9717-1 21136139

[B7] Bao G. Zhuo C. Qian C. Xiao T. Guo Z. Lu S. (2016). Co-expression of NCED and ALO improves vitamin C level and tolerance to drought and chilling in transgenic tobacco and stylo plants. Plant Biotechnol. J. 14, 206–214. 10.1111/pbi.12374 25865630 PMC11388907

[B8] Benson J. Zeevaart J. (1990). Comparison of ent-kaurene synthase A and B activities in cell-free extracts from young tomato fruits of wild-type and gib-1, gib-2, and gib-3 tomato plants. J. Plant Growth Regul. 9, 237–242. 10.1007/BF02041969

[B9] Bentsink L. Jowett J. Hanhart C. J. Koornneef M. (2006). Cloning of DOG1, a quantitative trait locus controlling seed dormancy in *Arabidopsis*. Proc. Natl. Acad. Sci. U.S.A. 103, 17042–17047. 10.1073/pnas.0607877103 17065317 PMC1636575

[B10] Bi C. Ma Y. Wang X.-F. Zhang D.-P. (2017). Overexpression of the transcription factor NF-YC9 confers abscisic acid hypersensitivity in *Arabidopsis*. Plant Mol. Biol. 95, 425–439. 10.1007/s11103-017-0661-1 28924726 PMC5688200

[B11] Burla B. Pfrunder S. Nagy R. Francisco R. M. Lee Y. Martinoia E. (2013). Vacuolar transport of abscisic acid glucosyl ester is mediated by ATP-binding cassette and proton-antiport mechanisms in *Arabidopsis*. Plant Physiol. 163, 1446–1458. 10.1104/pp.113.222547 24028845 PMC3813663

[B12] Chan Z. (2012). Expression profiling of ABA pathway transcripts indicates crosstalk between abiotic and biotic stress responses in *Arabidopsis*. Genomics 100, 110–115. 10.1016/j.ygeno.2012.06.004 22709556

[B13] Chen H. Y. Hsieh E. J. Cheng M. C. Chen C. Y. Hwang S. Y. Lin T. P. (2016). ORA47 (octadecanoid-responsive AP2/ERF-domain transcription factor 47) regulates jasmonic acid and abscisic acid biosynthesis and signaling through binding to a novel cis-element. New Phytol. 211, 599–613. 10.1111/nph.13914 26974851

[B14] Cheng W. H. Endo A. Zhou L. Penney J. Chen H. C. Arroyo A.. (2002). A unique short-chain dehydrogenase/reductase in *Arabidopsis* glucose signaling and abscisic acid biosynthesis and functions. Plant Cell 14, 2723–2743. 10.1105/tpc.006494 12417697 PMC152723

[B15] Chiu R. S. Nahal H. Provart N. J. Gazzarrini S. (2012). The role of the *Arabidopsis* FUSCA3 transcription factor during inhibition of seed germination at high temperature. BMC Plant Biol. 12:15 10.1186/1471-2229-12-15 22279962 PMC3296646

[B16] Choi H.-I. Hong J.-H. Ha J.-O. Kang J.-Y. Kim S. Y. (2000). ABFs, a family of ABA-responsive element binding factors. J. Biol. Chem. 275, 1723–1730. 10.1074/jbc.275.3.1723 10636868

[B17] Colebrook E. H. Thomas S. G. Phillips A. L. Hedden P. (2014). The role of gibberellin signalling in plant responses to abiotic stress. J. Exp. Biol. 217, 67–75. 10.1242/jeb.089938 24353205

[B18] Davière J.-M. Achard P. (2013). Gibberellin signaling in plants. Development 140, 1147–1151. 10.1242/dev.087650 23444347

[B19] Dey A. Samanta M. K. Gayen S. Maiti M. K. (2016). The sucrose non-fermenting 1-related kinase 2 gene SAPK9 improves drought tolerance and grain yield in rice by modulating cellular osmotic potential, stomatal closure and stress-responsive gene expression. BMC Plant Biol. 16:158 10.1186/s12870-016-0845-x 27411911 PMC4944446

[B20] Dong Z. Yu Y. Li S. Wang J. Tang S. Huang R. (2016). Abscisic acid antagonizes ethylene production through the ABI4-mediated transcriptional repression of ACS4 and ACS8 in *Arabidopsis*. Mol. Plant 9, 126–135. 10.1016/j.molp.2015.09.007 26410794

[B21] Dubois M. Skirycz A. Claeys H. Maleux K. Dhondt S. De Bodt S.. (2013). Ethylene Response Factor6 acts as a central regulator of leaf growth under water-limiting conditions in *Arabidopsis*. Plant Physiol. 162, 319–332. 10.1104/pp.113.216341 23553636 PMC3641212

[B22] Estrada-Melo A. C. Chao Reid M. S. Jiang C.-Z. (2015). Overexpression of an ABA biosynthesis gene using a stress-inducible promoter enhances drought resistance in petunia. Hortic. Res. 2:15013. 10.1038/hortres.2015.13 26504568 PMC4595983

[B23] Finkelstein R. R. (1994). Mutations at two new *Arabidopsis* ABA response loci are similar to the abi3 mutations. Plant J. 5, 765–771. 10.1046/j.1365-313X.1994.5060765.x

[B24] Finkelstein R. Reeves W. Ariizumi T. Steber C. (2008). Molecular aspects of seed dormancy. Annu. Rev. Plant Biol. 59, 387–415. 10.1146/annurev.arplant.59.032607.092740 18257711

[B25] Finkelstein R. R. Gampala S. S. Rock C. D. (2002). Abscisic acid signaling in seeds and seedlings. Plant Cell 14(Suppl.), S15–S45. 10.1105/tpc.010441 12045268 PMC151246

[B26] Finkelstein R. R. Lynch T. J. (2000). The *Arabidopsis* abscisic acid response gene ABI5 encodes a basic leucine zipper transcription factor. Plant Cell 12, 599–609. 10.1105/tpc.12.4.599 10760247 PMC139856

[B27] Fleet C. M. Sun T. P. (2005). A DELLAcate balance: the role of gibberellin in plant morphogenesis. Curr. Opin. Plant Biol. 8, 77–85. 10.1016/j.pbi.2004.11.015 15653404

[B28] Footitt S. Douterelo-Soler I. Clay H. Finch-Savage W. E. (2011). Dormancy cycling in *Arabidopsis* seeds is controlled by seasonally distinct hormone-signaling pathways. Proc. Natl. Acad. Sci. U.S.A. 108, 20236–20241. 10.1073/pnas.1116325108 22128331 PMC3250134

[B29] Fujita Y. Nakashima K. Yoshida T. Katagiri T. Kidokoro S. Kanamori N.. (2009). Three SnRK2 protein kinases are the main positive regulators of abscisic acid signaling in response to water stress in *Arabidopsis*. Plant Cell Physiol. 50, 2123–2132. 10.1093/pcp/pcp147 19880399

[B30] Fukao T. Bailey-Serres J. (2008). Submergence tolerance conferred by Sub1A is mediated by SLR1 and SLRL1 restriction of gibberellin responses in rice. Proc. Natl. Acad. Sci. U.S.A. 105, 16814–16819. 10.1073/pnas.0807821105 18936491 PMC2575502

[B31] Gabriele S. Rizza A. Martone J. Circelli P. Costantino P. Vittorioso P. (2010). The Dof protein DAG1 mediates PIL5 activity on seed germination by negatively regulating GA biosynthetic gene AtGA3ox1. Plant J. 61, 312–323. 10.1111/j.1365-313X.2009.04055.x 19874540

[B32] García-Martínez J. L. Lopez-Diaz I. Sanchez-Beltran M. J. Phillips A. L. Ward D. A. Gaskin P.. (1997). Isolation and transcript analysis of gibberellin 20-oxidase genes in pea and bean in relation to fruit development. Plant Mol. Biol. 33, 1073–1084. 10.1023/A:1005715722193 9154988

[B33] Gazzarrini S. Tsuchiya Y. Lumba S. Okamoto M. McCourt P. (2004). The transcription factor FUSCA3 controls developmental timing in *Arabidopsis* through the hormones gibberellin and abscisic acid. Dev. Cell 7, 373–385. 10.1016/j.devcel.2004.06.017 15363412

[B34] Gimeno-Gilles C. Lelièvre E. Viau L. Malik-Ghulam M. Ricoult C. Niebel A.. (2009). ABA-mediated inhibition of germination is related to the inhibition of genes encoding cell-wall biosynthetic and architecture: modifying enzymes and structural proteins in medicago truncatula embryo axis. Mol. Plant 2, 108–119. 10.1093/mp/ssn092 19529818 PMC2639729

[B35] González-Guzmán M. Rodriguez L. Lorenzo-Orts L. Pons C. Sarrion-Perdigones A. Fernandez M. A.. (2014). Tomato PYR/PYL/RCAR abscisic acid receptors show high expression in root, differential sensitivity to the abscisic acid agonist quinabactin, and the capability to enhance plant drought resistance. J. Exp. Bot. 65, 4451–4464. 10.1093/jxb/eru219 24863435 PMC4112642

[B36] Guo X. Hou X. Fang J. Wei P. Xu B. Chen M.. (2013). The rice GERMINATION DEFECTIVE 1, encoding a B3 domain transcriptional repressor, regulates seed germination and seedling development by integrating GA and carbohydrate metabolism. Plant J. 75, 403–416. 10.1111/tpj.12209 23581288 PMC3813988

[B37] Han C. Yang P. (2015). Studies on the molecular mechanisms of seed germination. Proteomics 15, 1671–1679. 10.1002/pmic.201400375 25597791

[B38] Hattori Y. Nagai K. Furukawa S. Song X. J. Kawano R. Sakakibara H.. (2009). The ethylene response factors SNORKEL1 and SNORKEL2 allow rice to adapt to deep water. Nature 460, 1026–1030. 10.1038/nature08258 19693083

[B39] Hauser F. Waadt R. Schroeder J. I. (2011). Evolution of abscisic acid synthesis and signaling mechanisms. Curr. Biol. 21, R346–355. 10.1016/j.cub.2011.03.015 21549957 PMC3119208

[B40] Hedden P. (2001). Gibberellin metabolism and its regulation. J. Plant Growth Regul. 20, 317–318. 10.1007/s003440010039 11986757

[B41] Hedden P. Phillips A. L. (2000). Gibberellin metabolism: new insights revealed by the genes. Trends Plant Sci. 5, 523–530. 10.1016/S1360-1385(00)01790-8 11120474

[B42] Hedden P. Thomas S. G. (2012). Gibberellin biosynthesis and its regulation. Biochem. J. 444, 11–25. 10.1042/BJ20120245 22533671

[B43] Heyman J. Cools T. Canher B. Shavialenka S. Traas J. Vercauteren I.. (2016). The heterodimeric transcription factor complex ERF115–PAT1 grants regeneration competence. Nat. Plants 2:16165. 10.1038/nplants.2016.165 27797356

[B44] Holdsworth M. J. Bentsink L. Soppe W. J. (2008). Molecular networks regulating *Arabidopsis* seed maturation, after-ripening, dormancy and germination. New Phytol. 179, 33–54. 10.1111/j.1469-8137.2008.02437.x 18422904

[B45] Hossain M. A. Cho J.-I. Han M. Ahn C.-H. Jeon J.-S. An G.. (2010). The ABRE-binding bZIP transcription factor OsABF2 is a positive regulator of abiotic stress and ABA signaling in rice. J. Plant Physiol. 167, 1512–1520. 10.1016/j.jplph.2010.05.008 20576316

[B46] Itoh H. Tanaka-Ueguchi M. Kawaide H. Chen X. Kamiya Y. Matsuoka M. (1999). The gene encoding tobacco gibberellin 3β-hydroxylase is expressed at the site of GA action during stem elongation and flower organ development. Plant J. 20, 15–24. 10.1046/j.1365-313X.1999.00568.x 10571861

[B47] Iwata A. Durai V. Tussiwand R. Briseño C. G. Wu X. Grajales-Reyes G. E. (2017). Quality of TCR signaling determined by differential affinities of enhancers for the composite BATF–IRF4 transcription factor complex. Nat. Immunol. 18:563 10.1038/ni.3714 28346410 PMC5401770

[B48] Kaneko M. Itoh H. Inukai Y. Sakamoto T. Ueguchi-Tanaka M. Ashikari M.. (2003). Where do gibberellin biosynthesis and gibberellin signaling occur in rice plants? Plant J. 35, 104–115. 10.1046/j.1365-313X.2003.01780.x 12834406

[B49] Kaneko M. Itoh H. Ueguchi-Tanaka M. Ashikari M. Matsuoka M. (2002). The α-amylase induction in endosperm during rice seed germination is caused by gibberellin synthesized in epithelium. Plant Physiol. 128, 1264–1270. 10.1104/pp.010785 11950975 PMC154254

[B50] Kang J. Hwang J.-U. Lee M. Kim Y.-Y. Assmann S. M. Martinoia E.. (2010). PDR-type ABC transporter mediates cellular uptake of the phytohormone abscisic acid. Proc. Natl. Acad. Sci. U.S.A. 107, 2355–2360. 10.1073/pnas.0909222107 20133880 PMC2836657

[B51] Kanno Y. Hanada A. Chiba Y. Ichikawa T. Nakazawa M. Matsui M.. (2012). Identification of an abscisic acid transporter by functional screening using the receptor complex as a sensor. Proc. Natl. Acad. Sci. U.S.A. 109, 9653–9658. 10.1073/pnas.1203567109 22645333 PMC3386071

[B52] Karssen C. Brinkhorst-Van Der Swan D. Breekland A. Koornneef M. (1983). Induction of dormancy during seed development by endogenous abscisic acid: studies on abscisic acid deficient genotypes of *Arabidopsis thaliana* (L.) Heynh. Planta 157, 158–165. 10.1007/BF00393650 24264070

[B53] Karssen C. M. Zagorski S. Kepczynski J. Groot S. (1989). Key role for endogenous gibberellins in the control of seed germination. Ann. Bot. 63, 71–80. 10.1093/oxfordjournals.aob.a087730

[B54] Keith K. Kraml M. Dengler N. G. McCourt P. (1994). fusca3: a heterochronic mutation affecting late embryo development in *Arabidopsis*. Plant Cell 6, 589–600. 10.1105/tpc.6.5.589 12244252 PMC160461

[B55] Kepka M. Benson C. L. Gonugunta V. K. Nelson K. M. Christmann A. Grill E.. (2011). Action of natural abscisic acid precursors and catabolites on abscisic acid receptor complexes. Plant Physiol. 157, 2108–2119. 10.1104/pp.111.182584 21976481 PMC3327214

[B56] Kim D. H. Yamaguchi S. Lim S. Oh E. Park J. Hanada A.. (2008). SOMNUS, a CCCH-type zinc finger protein in *Arabidopsis*, negatively regulates light-dependent seed germination downstream of PIL5. Plant Cell 20, 1260–1277. 10.1105/tpc.108.058859 18487351 PMC2438461

[B57] Kim S.-I. Park B. S. Yeu S. Y. Song S. I. Song J. T. Seo H. S. (2015). E3 SUMO ligase AtSIZ1 positively regulates SLY1-mediated GA signalling and plant development. Biochem. J. 469, 299–314. 10.1042/BJ20141302 26008766

[B58] Kim S. Y. Ma J. Perret P. Li Z. Thomas T. L. (2002). *Arabidopsis* ABI5 subfamily members have distinct DNA-binding and transcriptional activities. Plant Physiol. 130, 688–697. 10.1104/pp.003566 12376636 PMC166598

[B59] Ko J. H. Yang S. H. Han K. H. (2006). Upregulation of an *Arabidopsis* RING-H2 gene, XERICO, confers drought tolerance through increased abscisic acid biosynthesis. Plant J. 47, 343–355. 10.1111/j.1365-313X.2006.02782.x 16792696

[B60] Kobayashi Y. Murata M. Minami H. Yamamoto S. Kagaya Y. Hobo T.. (2005). Abscisic acid-activated SNRK2 protein kinases function in the gene-regulation pathway of ABA signal transduction by phosphorylating ABA response element-binding factors. Plant J. 44, 939–949. 10.1111/j.1365-313X.2005.02583.x 16359387

[B61] Kohli A. Sreenivasulu N. Lakshmanan P. Kumar P. P. (2013). The phytohormone crosstalk paradigm takes center stage in understanding how plants respond to abiotic stresses. Plant Cell Rep. 32, 945–957. 10.1007/s00299-013-1461-y 23749097

[B62] Koornneef M. Van Der Veen J. (1980). Induction and analysis of gibberellin sensitive mutants in *Arabidopsis thaliana* (L.) Heynh. Theor. Appl. Genet. 58, 257–263. 10.1007/BF00265176 24301503

[B63] Kumar P. P. (2013a). Regulation of biotic and abiotic stress responses by plant hormones. Plant Cell Rep. 32:943 10.1007/s00299-013-1460-z 23715739

[B64] Kumar P. P. (2013b). Plant hormones and their intricate signaling networks: unraveling the nexus. Plant Cell Rep. 32, 731–732. 10.1007/s00299-013-1435-0 23543367

[B65] Kuromori T. Miyaji T. Yabuuchi H. Shimizu H. Sugimoto E. Kamiya A.. (2010). ABC transporter AtABCG25 is involved in abscisic acid transport and responses. Proc. Natl. Acad. Sci. U.S.A. 107, 2361–2366. 10.1073/pnas.0912516107 20133881 PMC2836683

[B66] Kuromori T. Sugimoto E. Shinozaki K. (2011). *Arabidopsis* mutants of AtABCG22, an ABC transporter gene, increase water transpiration and drought susceptibility. Plant J. 67, 885–894. 10.1111/j.1365-313X.2011.04641.x 21575091

[B67] Lee H. G. Lee K. Seo P. J. (2015). The Arabidopsis MYB96 transcription factor plays a role in seed dormancy. Plant Mol. Biol. 87, 371–381. 10.1007/s11103-015-0283-4 25616734

[B68] Lee K. H. Piao H. L. Kim H.-Y. Choi S. M. Jiang F. Hartung W.. (2006). Activation of glucosidase via stress-induced polymerization rapidly increases active pools of abscisic acid. Cell 126, 1109–1120. 10.1016/j.cell.2006.07.034 16990135

[B69] Lee S. C. Lee M. Y. Kim S. J. Jun S. H. An G. Kim S. R. (2005). Characterization of an abiotic stress-inducible dehydrin gene, OsDhn1, in rice (*Oryza sativa L*.). Mol. Cells 19, 212–218. 15879704

[B70] Lee S. C. Luan S. (2012). ABA signal transduction at the crossroad of biotic and abiotic stress responses. Plant Cell Environ. 35, 53–60. 10.1111/j.1365-3040.2011.02426.x 21923759

[B71] Lefebvre V. North H. Frey A. Sotta B. Seo M. Okamoto M.. (2006). Functional analysis of *Arabidopsis* NCED6 and NCED9 genes indicates that ABA synthesized in the endosperm is involved in the induction of seed dormancy. Plant J. 45, 309–319. 10.1111/j.1365-313X.2005.02622.x 16412079

[B72] Li C. Li J. Chong K. Harter K. Lee Y. Leung J.. (2016). Toward a molecular understanding of plant hormone actions. Mol. Plant 9, 1–3. 10.1016/j.molp.2015.12.006 26708606

[B73] Lim E.-K. Doucet C. J. Hou B. Jackson R. G. Abrams S. R. Bowles D. J. (2005). Resolution of (+)-abscisic acid using an *Arabidopsis* glycosyltransferase. Tetrahedron 16, 143–147. 10.1016/j.tetasy.2004.11.062

[B74] Lim S. Park J. Lee N. Jeong J. Toh S. Watanabe A.. (2013). ABA-INSENSITIVE3, ABA-INSENSITIVE5, and DELLAs interact to activate the expression of SOMNUS and other high-temperature-inducible genes in imbibed seeds in *Arabidopsis*. Plant Cell 25, 4863–4878. 10.1105/tpc.113.118604 24326588 PMC3903992

[B75] Lin P. C. Hwang S. G. Endo A. Okamoto M. Koshiba T. Cheng W. H. (2007). Ectopic expression of ABSCISIC ACID 2/GLUCOSE INSENSITIVE 1 in *Arabidopsis* promotes seed dormancy and stress tolerance. Plant Physiol. 143, 745–758. 10.1104/pp.106.084103 17189333 PMC1803738

[B76] Lin Q. Wu F. Sheng P. Zhang Z. Zhang X. Guo X.. (2015). The SnRK2-APC/C TE regulatory module mediates the antagonistic action of gibberellic acid and abscisic acid pathways. Nat. Commun. 6:7981. 10.1038/ncomms8981 26272249 PMC4557272

[B77] Liu C. Mao B. Ou S. Wang W. Liu L. Wu Y.. (2014). OsbZIP71, a bZIP transcription factor, confers salinity and drought tolerance in rice. Plant Mol. Biol. 84, 19–36. 10.1007/s11103-013-0115-3 23918260

[B78] Liu C. Wu Y. Wang X. (2012). bZIP transcription factor OsbZIP52/RISBZ5: a potential negative regulator of cold and drought stress response in rice. Planta 235, 1157–1169. 10.1007/s00425-011-1564-z 22189955

[B79] Liu J. Mehdi S. Topping J. Tarkowski P. Lindsey K. (2010). Modelling and experimental analysis of hormonal crosstalk in *Arabidopsis*. Mol. Syst. Biol. 6:373. 10.1038/msb.2010.26 20531403 PMC2913391

[B80] Liu P. P. Koizuka N. Martin R. C. Nonogaki H. (2005). The BME3 (Blue Micropylar End 3) GATA zinc finger transcription factor is a positive regulator of *Arabidopsis* seed germination. Plant J. 44, 960–971. 10.1111/j.1365-313X.2005.02588.x 16359389

[B81] Liu X. Hou X. (2018). Antagonistic regulation of ABA and GA in metabolism and signaling pathways. Front. Plant Sci. 9:251. 10.3389/fpls.2018.00251 29535756 PMC5834473

[B82] Liu X. Hu P. Huang M. Tang Y. Li Y. Li L.. (2016). The NF-YC–RGL2 module integrates GA and ABA signalling to regulate seed germination in *Arabidopsis*. Nat. Commun. 7:12768. 10.1038/ncomms12768 27624486 PMC5027291

[B83] Lopez-Molina L. Chua N.-H. (2000). A null mutation in a bZIP factor confers ABA-insensitivity in *Arabidopsis thaliana*. Plant Cell Physiol. 41, 541–547. 10.1093/pcp/41.5.541 10929936

[B84] Lopez-Molina L. Mongrand S. McLachlin D. T. Chait B. T. Chua N. H. (2002). ABI5 acts downstream of ABI3 to execute an ABA-dependent growth arrest during germination. Plant J. 32, 317–328. 10.1046/j.1365-313X.2002.01430.x 12410810

[B85] Luo A. Qian Q. Yin H. Liu X. Yin C. Lan Y.. (2006). EUI1, encoding a putative cytochrome P450 monooxygenase, regulates internode elongation by modulating gibberellin responses in rice. Plant Cell Physiol. 47, 181–191. 10.1093/pcp/pci233 16306061

[B86] Ma Y. Szostkiewicz I. Korte A. Moes D. Yang Y. Christmann A.. (2009). Regulators of PP2C phosphatase activity function as abscisic acid sensors. Science 324, 1064–1068. 10.1126/science.1172408 19407143

[B87] Magome H. Yamaguchi S. Hanada A. Kamiya Y. Oda K. (2008). The DDF1 transcriptional activator upregulates expression of a gibberellin-deactivating gene, GA2ox7, under high-salinity stress in *Arabidopsis*. Plant J. 56, 613–626. 10.1111/j.1365-313X.2008.03627.x 18643985

[B88] Mahajan S. Tuteja N. (2005). Cold, salinity and drought stresses: an overview. Arch. Biochem. Biophys. 444, 139–158. 10.1016/j.abb.2005.10.018 16309626

[B89] Martínez-Andújar C. Ordiz M. I. Huang Z. Nonogaki M. Beachy R. N. Nonogaki H. (2011). Induction of 9-cis-epoxycarotenoid dioxygenase in *Arabidopsis* thaliana seeds enhances seed dormancy. Proc. Natl. Acad. Sci. U.S.A. 108, 17225–17229. 10.1073/pnas.1112151108 21969557 PMC3193201

[B90] Miura K. Lee J. Jin J. B. Yoo C. Y. Miura T. Hasegawa P. M. (2009). Sumoylation of ABI5 by the *Arabidopsis* SUMO E3 ligase SIZ1 negatively regulates abscisic acid signaling. Proc. Natl. Acad. Sci. U.S.A. 106, 5418–5423. 10.1073/pnas.0811088106 19276109 PMC2664011

[B91] Murase K. Hirano Y. Sun T.-P. Hakoshima T. (2008). Gibberellin-induced DELLA recognition by the gibberellin receptor GID1. Nature 456, 459. 10.1038/nature07519 19037309

[B92] Ng L. M. Melcher K. Teh B. T. Xu H. E. (2014). Abscisic acid perception and signaling: structural mechanisms and applications. Acta Pharmacol. Sin. 35:567 10.1038/aps.2014.5 24786231 PMC4813750

[B93] Nir I. Shohat H. Panizel I. Olszewski N. Aharoni A. Weiss D. (2017). The tomato DELLA protein PROCERA acts in guard cells to promote stomatal closure. Plant Cell 29, 3186–3197. 10.1105/tpc.17.00542 29150547 PMC5757276

[B94] Oh E. Yamaguchi S. Hu J. Yusuke J. Jung B. Paik I.. (2007). PIL5, a phytochrome-interacting bHLH protein, regulates gibberellin responsiveness by binding directly to the GAI and RGA promoters in *Arabidopsis* seeds. Plant Cell 19, 1192–1208. 10.1105/tpc.107.050153 17449805 PMC1913757

[B95] Oh E. Yamaguchi S. Kamiya Y. Bae G. Chung W. I. Choi G. (2006). Light activates the degradation of PIL5 protein to promote seed germination through gibberellin in *Arabidopsis*. Plant J. 47, 124–139. 10.1111/j.1365-313X.2006.02773.x 16740147

[B96] Park J. Lee N. Kim W. Lim S. Choi G. (2011). ABI3 and PIL5 collaboratively activate the expression of SOMNUS by directly binding to its promoter in imbibed *Arabidopsis* seeds. Plant Cell 23, 1404–1415. 10.1105/tpc.110.080721 21467583 PMC3101561

[B97] Park S. Y. Fung P. Nishimura N. Jensen D. R. Fujii H. Zhao Y.. (2009). Abscisic acid inhibits type 2C protein phosphatases via the PYR/PYL family of START proteins. Science 324, 1068–1071. 10.1126/science.1173041 19407142 PMC2827199

[B98] Peirats-Llobet M. Han S.-K. Gonzalez-Guzman M. Jeong C. W. Rodriguez L. Belda-Palazon B.. (2016). A direct link between abscisic acid sensing and the chromatin-remodeling ATPase BRAHMA via core ABA signaling pathway components. Mol. Plant 9, 136–147. 10.1016/j.molp.2015.10.003 26499068

[B99] Penfield S. Josse E.-M. Kannangara R. Gilday A. D. Halliday K. J. Graham I. A. (2005). Cold and light control seed germination through the bHLH transcription factor SPATULA. Curr. Biol. 15, 1998–2006. 10.1016/j.cub.2005.11.010 16303558

[B100] Peng J. Carol P. Richards D. E. King K. E. Cowling R. J. Murphy G. P.. (1997). The *Arabidopsis* GAI gene defines a signaling pathway that negatively regulates gibberellin responses. Genes Dev. 11, 3194–3205. 10.1101/gad.11.23.3194 9389651 PMC316750

[B101] Peng J. Richards D. E. Hartley N. M. Murphy G. P. Devos K. M. Flintham J. E.. (1999). ‘Green revolution' genes encode mutant gibberellin response modulators. Nature 400, 256–261. 10.1038/22307 10421366

[B102] Qin F. Kodaira K. S. Maruyama K. Mizoi J. Tran L. S. Fujita Y.. (2011). SPINDLY, a negative regulator of gibberellic acid signaling, is involved in the plant abiotic stress response. Plant Physiol. 157, 1900–1913. 10.1104/pp.111.187302 22013217 PMC3327212

[B103] Rajjou L. Duval M. Gallardo K. Catusse J. Bally J. Job C.. (2012). Seed germination and vigor. Annu. Rev. Plant Biol. 63, 507–533. 10.1146/annurev-arplant-042811-105550 22136565

[B104] Ravindran P. Verma V. Stamm P. Kumar P. P. (2017). A novel RGL2-DOF6 complex contributes to primary seed dormancy in *Arabidopsis thaliana* by regulating a GATA transcription factor. Mol. Plant 10, 1307–1320. 10.1016/j.molp.2017.09.004 28917589

[B105] Rowe J. H. Topping J. F. Liu J. Lindsey K. (2016). Abscisic acid regulates root growth under osmotic stress conditions via an interacting hormonal network with cytokinin, ethylene and auxin. New Phytol. 211, 225–239. 10.1111/nph.13882 26889752 PMC4982081

[B106] Ruiz-Sola M. A. Rodriguez-Concepcion M. (2012). Carotenoid biosynthesis in *Arabidopsis*: a colorful pathway. Arabidopsis Book 10:e0158. 10.1199/tab.0158 22582030 PMC3350171

[B107] Ryu H. Cho H. Bae W. Hwang I. (2014). Control of early seedling development by BES1/TPL/HDA19-mediated epigenetic regulation of ABI3. Nat. Commun. 5:4138. 10.1038/ncomms5138 24938150

[B108] Saavedra X. Modrego A. Rodriguez D. Gonzalez-Garcia M. P. Sanz L. Nicolas G.. (2010). The nuclear interactor PYL8/RCAR3 of Fagus sylvatica FsPP2C1 is a positive regulator of abscisic acid signaling in seeds and stress. Plant Physiol. 152, 133–150. 10.1104/pp.109.146381 19889877 PMC2799352

[B109] Sah S. K. Reddy K. R. Li J. (2016). Abscisic acid and abiotic stress tolerance in crop plants. Front. Plant Sci. 7:571. 10.3389/fpls.2016.00571 27200044 PMC4855980

[B110] Santiago J. Dupeux F. Round A. Antoni R. Park S. Y. Jamin M.. (2009). The abscisic acid receptor PYR1 in complex with abscisic acid. Nature 462, 665–668. 10.1038/nature08591 19898494

[B111] Santner A. Calderon-Villalobos L. I. Estelle M. (2009). Plant hormones are versatile chemical regulators of plant growth. Nat. Chem. Biol. 5, 301–307. 10.1038/nchembio.165 19377456

[B112] Schopfer P. Plachy C. (1984). Control of seed germination by abscisic acid. Plant Physiol. 76, 155–160. 10.1104/pp.76.1.155 16663788 PMC1064247

[B113] Sharkey T. D. Raschke K. (1980). Effects of phaseic Acid and dihydrophaseic Acid on stomata and the photosynthetic apparatus. Plant Physiol. 65, 291–297. 10.1104/pp.65.2.291 16661176 PMC440313

[B114] Shu K. Chen Q. Wu Y. Liu R. Zhang H. Wang P.. (2016). ABI4 mediates antagonistic effects of abscisic acid and gibberellins at transcript and protein levels. The Plant Journal 85, 348–361. 10.1111/tpj.13109 26708041

[B115] Shu K. Zhang H. Wang S. Chen M. Wu Y. Tang S.. (2013). ABI4 regulates primary seed dormancy by regulating the biogenesis of abscisic acid and gibberellins in *Arabidopsis*. PLoS Genet. 9:e1003577. 10.1371/journal.pgen.1003577 23818868 PMC3688486

[B116] Shu K. Zhou W. Yang W. (2018). APETALA 2-domain-containing transcription factors: focusing on abscisic acid and gibberellins antagonism. New Phytol. 217, 977–983. 10.1111/nph.14880 29058311

[B117] Silverstone A. L. Chang C. W. Krol E. Sun T. P. (1997). Developmental regulation of the gibberellin biosynthetic gene GA1 in *Arabidopsis thaliana*. Plant J. 12, 9–19. 10.1046/j.1365-313X.1997.12010009.x 9263448

[B118] Skubacz A. Daszkowska-Golec A. (2017). Seed dormancy: the complex process regulated by abscisic acid, gibberellins, and other phytohormones that makes seed germination work, in Phytohormones - Signaling Mechanisms and Crosstalk in Plant Development and Stress Responses, ed El-Esawi M. (InTech), 77–100. 10.5772/intechopen.68735

[B119] Song L. Huang S. C. Wise A. Castanon R. Nery J. R. Chen H.. (2016). A transcription factor hierarchy defines an environmental stress response network. Science 354:aag1550. 10.1126/science.aag1550 27811239 PMC5217750

[B120] Stamm P. Kumar P. P. (2013). Auxin and gibberellin responsive *Arabidopsis* SMALL AUXIN UP RNA36 regulates hypocotyl elongation in the light. Plant Cell Rep. 32, 759–769. 10.1007/s00299-013-1406-5 23503980

[B121] Stamm P. Ravindran P. Mohanty B. Tan E. L. Yu H. Kumar P. P. (2012). Insights into the molecular mechanism of RGL2-mediated inhibition of seed germination in *Arabidopsis* thaliana. BMC Plant Biol. 12:179 10.1186/1471-2229-12-179 23035751 PMC3732085

[B122] Steber C. M. Cooney S. E. Mccourt P. (1998). Isolation of the GA-response mutant sly1 as a suppressor of ABI1-1 in *Arabidopsis thaliana*. Genetics 149, 509–521. 9611170 10.1093/genetics/149.2.509 PMC1460187

[B123] Tang N. Zhang H. Li X. Xiao J. Xiong L. (2012). Constitutive activation of transcription factor OsbZIP46 improves drought tolerance in rice. Plant Physiol. 158, 1755–1768. 10.1104/pp.111.190389 22301130 PMC3320183

[B124] Toh S. Imamura A. Watanabe A. Nakabayashi K. Okamoto M. Jikumaru Y.. (2008). High temperature-induced abscisic acid biosynthesis and its role in the inhibition of gibberellin action in *Arabidopsis* seeds. Plant Physiol. 146, 1368–1385. 10.1104/pp.107.113738 18162586 PMC2259091

[B125] Vaistij F. E. Gan Y. Penfield S. Gilday A. D. Dave A. He Z.. (2013). Differential control of seed primary dormancy in *Arabidopsis* ecotypes by the transcription factor SPATULA. Proc. Natl. Acad. Sci. U.S.A. 110, 10866–10871. 10.1073/pnas.1301647110 23754415 PMC3696787

[B126] Varbanova M. Yamaguchi S. Yang Y. McKelvey K. Hanada A. Borochov R.. (2007). Methylation of gibberellins by *Arabidopsis* GAMT1 and GAMT2. Plant Cell 19, 32–45. 10.1105/tpc.106.044602 17220201 PMC1820973

[B127] Verma V. Ramamoorthy R. Kohli A. Kumar P. P. (2015). Rice research to break yield barriers. COSMOS 11, 37–54. 10.1142/S0219607715500032

[B128] Verma V. Ravindran P. Kumar P. P. (2016). Plant hormone-mediated regulation of stress responses. BMC Plant Biol. 16:86 10.1186/s12870-016-0771-y 27079791 PMC4831116

[B129] Verslues P. E. Bray E. A. (2006). Role of abscisic acid (ABA) and Arabidopsis thaliana ABA-insensitive loci in low water potential-induced ABA and proline accumulation. J. Exp. Bot. 57, 201–212. 10.1093/jxb/erj026 16339784

[B130] Vu N.-T. Kang H.-M. Kim Y.-S. Choi K.-Y. Kim I.-S. (2015). Growth, physiology, and abiotic stress response to abscisic acid in tomato seedlings. Hortic. Environ. Biotechnol. 56, 294–304. 10.1007/s13580-015-0106-1

[B131] Wang F. Zhu D. Huang X. Li S. Gong Y. Yao Q.. (2009). Biochemical insights on degradation of *Arabidopsis* DELLA proteins gained from a cell-free assay system. Plant Cell 21, 2378–2390. 10.1105/tpc.108.065433 19717618 PMC2751948

[B132] Wang P. Liu H. Hua H. Wang L. Song C.-P. (2011). A vacuole localized β-glucosidase contributes to drought tolerance in *Arabidopsis*. Chin. Sci. Bull. 56, 3538–3546. 10.1007/s11434-011-4802-7

[B133] Wilkinson S. Davies W. J. (2010). Drought, ozone, ABA and ethylene: new insights from cell to plant to community. Plant Cell Environ. 33, 510–525. 10.1111/j.1365-3040.2009.02052.x 19843256

[B134] Willige B. C. Ghosh S. Nill C. Zourelidou M. Dohmann E. M. Maier A.. (2007). The DELLA domain of GA INSENSITIVE mediates the interaction with the GA INSENSITIVE DWARF1A gibberellin receptor of *Arabidopsis*. Plant Cell 19, 1209–1220. 10.1105/tpc.107.051441 17416730 PMC1913748

[B135] Xi W. Liu C. Hou X. Yu H. (2010). MOTHER OF FT AND TFL1 regulates seed germination through a negative feedback loop modulating ABA signaling in *Arabidopsis*. Plant Cell 22, 1733–1748. 10.1105/tpc.109.073072 20551347 PMC2910974

[B136] Xiang Y. Tang N. Du H. Ye H. Xiong L. (2008). Characterization of OsbZIP23 as a key player of the basic leucine zipper transcription factor family for conferring abscisic acid sensitivity and salinity and drought tolerance in rice. Plant Physiol. 148, 1938–1952. 10.1104/pp.108.128199 18931143 PMC2593664

[B137] Xiong L. Lee H. Ishitani M. Zhu J. K. (2002). Regulation of osmotic stress-responsive gene expression by the LOS6/ABA1 locus in *Arabidopsis*. J. Biol. Chem. 277, 8588–8596. 10.1074/jbc.M109275200 11779861

[B138] Xu Z.-Y. Lee K. H. Dong T. Jeong J. C. Jin J. B. Kanno Y.. (2012). A vacuolar β-glucosidase homolog that possesses glucose-conjugated abscisic acid hydrolyzing activity plays an important role in osmotic stress responses in *Arabidopsis*. Plant Cell 24, 2184–2199. 10.1105/tpc.112.095935 22582100 PMC3442595

[B139] Yabuta T. Sumiki Y. (1938). On the crystal of gibberellin, a substance to promote plant growth. J. Agric. Chem. Soc. Jpn 14:1526 10.1271/nogeikagaku1924.14.12_1526

[B140] Yaish M. W. El-Kereamy A. Zhu T. Beatty P. H. Good A. G. Bi Y.-M.. (2010). The APETALA-2-like transcription factor OsAP2-39 controls key interactions between abscisic acid and gibberellin in rice. PLoS Genet. 6:e1001098. 10.1371/journal.pgen.1001098 20838584 PMC2936520

[B141] Yamaguchi S. (2008). Gibberellin metabolism and its regulation. Annu. Rev. Plant Biol. 59, 225–251. 10.1146/annurev.arplant.59.032607.092804 18173378

[B142] Yamaguchi S. Kamiya Y. (2000). Gibberellin biosynthesis: its regulation by endogenous and environmental signals. Plant Cell Physiol. 41, 251–257. 10.1093/pcp/41.3.251 10805587

[B143] Yamaguchi S. Kamiya Y. Sun T. P. (2001). Distinct cell-specific expression patterns of early and late gibberellin biosynthetic genes during *Arabidopsis* seed germination. Plant J. 28, 443–453. 10.1046/j.1365-313X.2001.01168.x 11737781

[B144] Yano R. Kanno Y. Jikumaru Y. Nakabayashi K. Kamiya Y. Nambara E. (2009). CHOTTO1, a putative double APETALA2 repeat transcription factor, is involved in abscisic acid-mediated repression of gibberellin biosynthesis during seed germination in *Arabidopsis*. Plant Physiol. 151, 641–654. 10.1104/pp.109.142018 19648230 PMC2754653

[B145] Ye N. Jia L. Zhang J. (2012). ABA signal in rice under stress conditions. Rice (N. Y). 5:1. 10.1186/1939-8433-5-1 24764501 PMC3834477

[B146] Ying S. Zhang D. F. Li H. Y. Liu Y. H. Shi Y. S. Song Y. C.. (2011). Cloning and characterization of a maize SnRK2 protein kinase gene confers enhanced salt tolerance in transgenic *Arabidopsis*. Plant Cell Rep. 30, 1683–1699. 10.1007/s00299-011-1077-z 21638061

[B147] Zeevaart J. A. D. Creelman R. A. (1988). Metabolism and physiology of abscisic acid. Annu. Rev. Plant Physiol. Plant Mol. Biol. 39, 439–473. 10.1146/annurev.pp.39.060188.002255

[B148] Zeng D.-E. Hou P. Xiao F. Liu Y. (2015). Overexpression of *Arabidopsis* XERICO gene confers enhanced drought and salt stress tolerance in rice (*Oryza Sativa* L.). J. Plant Biochem. Biotechnol. 24, 56–64. 10.1007/s13562-013-0236-4

[B149] Zentella R. Zhang Z. L. Park M. Thomas S. G. Endo A. Murase K.. (2007). Global analysis of della direct targets in early gibberellin signaling in *Arabidopsis*. Plant Cell 19, 3037–3057. 10.1105/tpc.107.054999 17933900 PMC2174696

[B150] Zhang J. Schurr U. Davies W. J. (1987). Control of stomatal behaviour by abscisic acid which apparently originates in the roots. J. Exp. Bot. 38, 1174–1181. 10.1093/jxb/38.7.1174

[B151] Zhang H. Zhu H. Pan Y. Yu Y. Luan S. Li L. (2014). A DTX/MATE-type transporter facilitates abscisic acid efflux and modulates ABA sensitivity and drought tolerance in *Arabidopsis*. Mol. Plant 7, 1522–1532. 10.1093/mp/ssu063 24851876

[B152] Zheng J. Chen F. Wang Z. Cao H. Li X. Deng X.. (2012). A novel role for histone methyltransferase KYP/SUVH4 in the control of *Arabidopsis* primary seed dormancy. New Phytol. 193, 605–616. 10.1111/j.1469-8137.2011.03969.x 22122546

[B153] Zhu J. K. (2002). Salt and drought stress signal transduction in plants. Annu. Rev. Plant Biol. 53, 247–273. 10.1146/annurev.arplant.53.091401.143329 12221975 PMC3128348

[B154] Zhu Y. Nomura T. Xu Y. Zhang Y. Peng Y. Mao B.. (2006). ELONGATED UPPERMOST INTERNODE encodes a cytochrome P450 monooxygenase that epoxidizes gibberellins in a novel deactivation reaction in rice. Plant Cell 18, 442–456. 10.1105/tpc.105.038455 16399803 PMC1356550

